# Cowden Syndrome and Concomitant Pulmonary Neuroendocrine Tumor: A Presentation of Two Cases

**DOI:** 10.1155/2015/265786

**Published:** 2015-12-22

**Authors:** Seppo W. Langer, Lene Ringholm, Christine I. Dali, Rene Horsleben Petersen, Åse Krogh Rasmussen, Anne-Marie Gerdes, Birgitte Federspiel, Ulrich Peter Knigge

**Affiliations:** ^1^Department of Oncology, ENETS Neuroendocrine Tumor Centre of Excellence, Rigshospitalet, Copenhagen University Hospital, 2100 Copenhagen, Denmark; ^2^Department of Medical Endocrinology, ENETS Neuroendocrine Tumor Centre of Excellence, Rigshospitalet, Copenhagen University Hospital, 2100 Copenhagen, Denmark; ^3^Department of Clinical Genetics, ENETS Neuroendocrine Tumor Centre of Excellence, Rigshospitalet, Copenhagen University Hospital, 2100 Copenhagen, Denmark; ^4^Department of Cardiothoracic Surgery, ENETS Neuroendocrine Tumor Centre of Excellence, Rigshospitalet, Copenhagen University Hospital, 2100 Copenhagen, Denmark; ^5^Department of Pathology, ENETS Neuroendocrine Tumor Centre of Excellence, Rigshospitalet, Copenhagen University Hospital, 2100 Copenhagen, Denmark; ^6^Department of Surgical Gastroenterology, ENETS Neuroendocrine Tumor Centre of Excellence, Rigshospitalet, Copenhagen University Hospital, 2100 Copenhagen, Denmark

## Abstract

Cowden Syndrome is a rare autosomal dominantly inherited disorder. Patients with Cowden Syndrome are at increased risk of various benign and malignant neoplasms in breast, endometrium, thyroid, gastrointestinal tract, and genitourinary system. Neuroendocrine tumors are ubiquitous neoplasms that may occur anywhere in the human body. Bronchopulmonary neuroendocrine tumors include four different histological subtypes, among these, typical and atypical pulmonary carcinoids. No association between Cowden Syndrome and neuroendocrine tumors has previously been described. We present two cases of Cowden Syndrome that were diagnosed with pulmonary carcinoids.

## 1. Introduction

Cowden Syndrome is a rare autosomal dominantly inherited multisystem disorder with a prevalence of 1 in 200,000–250,000 [1]. It is characterized by dysfunctional cell growth and apoptosis due to germline mutations of the PTEN tumor suppressor gene [1, 2]. Rare cases with mutations in the succinate dehydrogenase complex and in DNA-binding proteins have also been described [3]. The clinical features of Cowden Syndrome include macrocephaly (head circumference ≥97th percentile), benign mucocutaneous manifestations, Lhermitte-Duclos disease (dysplastic gangliocytoma of the cerebellum), and hamartomatous growths in the intestinal tract [1, 2, 4–6]. Patients with Cowden Syndrome are at increased risk of various benign and malignant neoplasms in breast, endometrium, thyroid, gastrointestinal tract, and genitourinary system [1, 2, 4–6].

Neuroendocrine tumors (NETs) are ubiquitous neoplasms that may occur anywhere in the human body [7, 8]. Pulmonary NETs include four different histological subtypes, namely, typical or atypical carcinoid, large-cell neuroendocrine carcinoma, and small-cell lung carcinoma [9]. Typical and atypical carcinoids are characterized by a tumor size >5 mm with carcinoid morphology and immunohistochemical expression of the cell markers chromogranin A and synaptophysin. Typical carcinoids have <2 mitoses/2 mm^2^, lack of necrosis, are often centrally localized, and metastasize in 5–20%, whereas atypical carcinoids have 2–10 mitoses/2 mm^2^ and/or necrosis, tend to be larger, are more commonly located peripherally in the lungs, and metastasize in 30–70% [10].

No association between Cowden Syndrome and NETs has previously been described. We present two cases of Cowden Syndrome, which were diagnosed with pulmonary NETs.

## 2. Case 1

A Caucasian woman born in 1959 was in 2012 diagnosed with Cowden Syndrome and a germline PTEN mutation in terms of a deletion of one nucleotide in exon 8 causing a frame shift (c.1009delT). The same mutation was revealed in a daughter with clinical signs of Cowden Syndrome and in two grandchildren with macrocephaly, autism, strabismus, and developmental delay especially concerning language and gross motor function. Family members were not systematically screened for NETs. The patient had no smoking history. Due to multiple adenomas in an atoxic nodular goiter subtotal thyroidectomy was performed in 2001 and again in 2013 due to recurrence. Pathological examination showed colloid nodular goiter and follicular adenoma. In 2001, she underwent a left mastectomy with axillary node dissection due to invasive ductal carcinoma with metastases to eight of 12 lymph nodes. In 2013, she had a right sided mastectomy because of invasive ductal carcinoma (estrogen receptor positive/HER2 negative) with metastasis to 1 of 17 axillary lymph nodes. On both occasions, adjuvant chemotherapy with cyclophosphamide, epirubicin, and 5-fluorouracil was given followed by hormonal therapy with tamoxifen. External irradiation was given after surgery in 2001, but not in 2013. In 2013, a FDG PET/CT scan revealed four suspicious tumors, two in each of the lungs. The two tumors in the lower lobe of the left lung were removed by a wedge resection. The histology read two typical carcinoids. Immunohistochemical staining was positive for chromogranin, synaptophysin, TTF-1, and CD56, while being negative for mammaglobin, GCDFP-15, estrogen receptor, p63, and cytokeratin 5/6. The mitotic count was <2/2 mm^2^ and tumor necrosis was absent. PTEN immunostaining showed a positive cytoplasmic reaction. By fluorescence in situ hybridization (FISH) analysis, the PTEN probe showed a ratio of 1.10 between gene and centromere and contained 1.74 PTEN genes per cell, which indicates normal conditions. No additional germline mutation analyses were performed. After surgery, the plasma chromogranin A was 38 pmol/L (reference 30–130) and two 24-hour samples of urinary 5-hydroxyindoleacetic acid were also normal. A postoperative 68Ga-DOTATOC PET/CT scan revealed different uptake in one of the tumors in the right lung. A lobectomy of the upper right lobe was performed. Histology showed a sclerosing hemangioma.

No sign of recurrence has been found 2 years after the resection of the typical carcinoids.

## 3. Case 2

A male Caucasian born in 1953 was diagnosed with Cowden Syndrome and a PTEN germline mutation in 2010. The mutation was a missense mutation (c.389G>A) in exon 5 causing an arginine-glutamine substitution. The mutation is located in a conserved active site. There was no family history of Cowden Syndrome and the patient had no children. No family members wanted genetic testing and they were not systematically screened for NETs. The patient had no smoking history.

The patient had a history of Lhermitte-Duclos disease, esophageal polyps, several hamartomas, and mucocutaneous manifestations. He successfully underwent surgery for malignant melanoma level 1 on the truncus in 2001. A total thyroidectomy for follicular thyroid carcinoma was performed in 2000.

In 2013, the patient was diagnosed with a 15 × 15 mm atypical carcinoid in the left lung that stained positive for chromogranin and synaptophysin and negative for serotonin. The mitotic count was 2/2 mm^2^. PTEN immunostaining showed a positive cytoplasmic reaction. By FISH analysis, the PTEN probe showed a ratio of 0.57 between gene and centromere and contained 1.16 PTEN genes per cell, which indicates a deletion. No additional germline mutation analyses were performed. Plasma chromogranin A was increased at 330–370 pmol/L (reference 30–130). The patient was on proton pump inhibitor therapy and had normal kidney function. A 68Ga-DOTATOC PET/CT scan revealed an uptake equivalent to physiological liver uptake in a hilar tumor of the left lung and metastases in the liver and bones. The tumor was classified as cT1aN1M1b. Palliative external radiation was given to osteolytic hipbone lesions. Temozolomide therapy was initiated, but due to progression, therapy later was changed to streptozotocin and fluorouracil and is ongoing.

## 4. Discussion

This case presentation describes two patients with Cowden Syndrome and germline PTEN mutations who were diagnosed with pulmonary neuroendocrine tumors. Both patients had the characteristic phenotype of Cowden Syndrome [1, 2, 4–6]. Case 1 had breast cancer, macrocephaly, thyroid adenoma, and a positive family history of Cowden Syndrome. Case 2 with Lhermitte-Duclos disease, thyroid cancer, skin melanoma, and gastrointestinal hamartomas is most likely a de novo mutation as seen in 10–45% of patients with Cowden Syndrome [11]. The finding of a positive cytoplasmic reaction by PTEN immunostaining in both cases supports an association between Cowden Syndrome and pulmonary neuroendocrine tumor. In Case 1, a normal ratio of PTEN genes in the tumor cells indicates normal conditions but does not rule out a frame shift mutation. In Case 2, a low ratio of PTEN genes in the tumor cells supports the finding of a deletion.

In a retrospective study of 156 PTEN germline mutation carriers, a small intestine carcinoid was diagnosed incidentally in one patient while a lung carcinoid was identified in another patient at autopsy [12]. The specific PTEN mutations in these two cases were not stated.

It is unknown if there is a correlation between genotype and phenotype for development of NETs. Our cases presented with different types of PTEN mutations, a frameshift mutation (Case 1) and a missense mutation (Case 2).

NETs have been considered a rare type of cancer, but from 1973 to 2004, the incidence has increased fivefold, from 1.09 to 5.25/100,000 [8]. Contributing factors may be pathologists' increased focus on the diagnosis and tumor classification and improved diagnostic tools as immunohistochemistry, plasma markers, and specific imaging for NET [8]. Among Caucasians, approximately 30% of NETs occur in the lungs as typical or atypical pulmonary carcinoid [8] with typical carcinoids presenting in 84% of cases [13]. NETs are often diagnosed incidentally. As seen in the two presented cases the pulmonary NETs were identified while examining the patients for other diseases. Plasma chromogranin A, commonly used for the clinical monitoring of NETs [14], was normal in Case 1 after resection of the carcinoid tumor and elevated in Case 2 with residual tumor and proton pump inhibitor therapy.

Cowden Syndrome is an often-overlooked cancer predisposition syndrome with large variability of clinical features, and many of the symptoms and signs are common in the general population. Therefore, Cowden Syndrome is likely to be underdiagnosed [2].

Intragenic or promoter PTEN mutations have been detected in up to 85% of patients with Cowden Syndrome [15, 16]. Cowden Syndrome is associated with loss-of-function mutations in the tumor suppressor gene PTEN, a component of the mammalian target of rapamycin (mTOR) pathway that plays a central role in controlling cell growth, proliferation, and metabolism [15]. High levels of dysregulated mTOR activity have been associated with PTEN-related diseases including Cowden Syndrome [17]. Animal studies suggest that pharmacological mTOR inhibition may promote regression of advanced mucocutaneous Cowden's disease-like lesions [18] and inhibit tumor growth of pulmonary carcinoid [19]. According to an in vitro study, mTOR inhibition may be effective in the treatment of pulmonary neuroendocrine tumors [20]. Prolonged progression free survival with mTOR inhibition and octreotide long-acting repeatable was seen in a placebo-controlled phase III trial among patients with advanced pulmonary NETs [21].

Long-term follow-up is warranted to establish whether patients with Cowden Syndrome are at increased risk for developing NETs or whether the findings presented here were incidental. Furthermore, studies are needed to explore whether mTOR inhibition represents a therapeutic option for the treatment of both Cowden Syndrome and pulmonary carcinoid NETs. Until such data become available, we recommend that patients with Cowden Syndrome, as part of their routine control [5], have an annual chromogranin A measurement to screen for NET development. If repeated, chromogranin A levels remain elevated without other explanations; somatostatin receptor imaging, for example, 68Ga-DOTATOC PET/CT, may be an option.
